# Liver Involvement in SARS-CoV-2 Vertically Infected Newborn: A Case Report

**DOI:** 10.3389/fped.2021.701722

**Published:** 2021-11-11

**Authors:** Ilaria Stolfi, Maria Giulia Conti, Alessandra Marciano, Lucia Dito, Fabio Natale, Monica Bartolucci, Raffaella Cellitti, Daniela Regoli, Alessandra Ticchiarelli, Ida Pangallo, Federica Pagano, Camilla Ajassa, Roberto Brunelli, Gianluca Terrin

**Affiliations:** ^1^Department of Maternal and Child Health, Sapienza University of Rome, Rome, Italy; ^2^Department of Molecular Medicine, Sapienza University of Rome, Rome, Italy; ^3^Department of Public Health and Infectious Diseases, Sapienza University of Rome, Rome, Italy

**Keywords:** neonatology, infectious diseases, COVID-19, liver function, vertical transmission

## Abstract

Neonatal SARS-CoV-2 infection can occur antenatally, peripartum, or postnatally. In the newborn, clinical manifestations may vary including fever and respiratory, gastrointestinal and neurological symptoms. Most commonly, they are subclinical. We herein present a case of vertical transmission of SARS-CoV-2 presenting with liver injury, characterized by an increase in serum transaminases.

## Introduction

The novel severe acute respiratory syndrome coronavirus 2 (SARS-CoV-2), which causes the disease termed coronavirus disease 2019 (COVID-19), was declared a pandemic on March 2020 (1).

The main clinical manifestations of COVID-19 involve the upper and lower respiratory system; however, it was demonstrated that other organs and systems might be implicated, including the liver and the gastrointestinal (GI) tract (2).

Recent studies suggest that children are less likely to become infected with the virus compared to adults (3, 4). In addition, newborns and infants have clinical symptoms and laboratory and radiologic abnormalities less specific and less evident compared to older individuals (5). Therefore, many cases might remain subclinical or unrecognized in early life, due to neonatal stronger innate immune response and lower propensity to proinflammatory cytokine response (6).

Hepatic injury in COVID-19 adults and children has been reported (2, 6–9). Liver injury was characterized by slight increases in hepatocyte-related enzymes, including alanine aminotransferase (ALT) and aspartate aminotransferase (AST). In children with COVID-19, hepatitis has been reported to be associated with a severe presentation of the disease named multisystem inflammatory syndrome in children (MIS-C) (7).

To our knowledge, hepatic involvement in SARS-CoV-2-infected term-born newborns has never been described. A recent report of the World Health Organization (WHO) defines the modality of mother-to-child transmission of SARS-CoV-2 (10). It mainly occurs horizontally in the early postnatal period, i.e., *via* droplets, respiratory secretions, saliva, and direct contact, but oro-fecal transmission is also described (11). Vertical transmission, i.e., *in utero* or intrapartum, is also possible (12, 13). The consequences of the vertical transmission on the fetus and newborn are still poorly defined.

We present a case of vertical transmission of SARS-CoV-2 infection in a newborn with hepatic injury.

## Case Report

A 20-year-old, gravida 1, para 0 was admitted to the University Hospital Policlinico Umberto I in < city>Rome < /city>, Italy, at 41 weeks of gestation with fever (T max 39.6°C) for 2 days before hospitalization. Real-time polymerase chain reaction (RT-PCR) for RdRp/N genes performed on nasopharyngeal swab sample, upon admission, yielded a positive result.

Pregnancy was referred uneventful, and all the ultrasound examinations and routine tests were normal until the diagnosis of COVID-19. The medical history of the woman was negative.

Thrombocytopenia (platelets (PLT) 127 ×10^3^/μl, normal range 150–450 × 10^3^/μl) and elevated C-reactive protein (CRP) 13,000 μg/l (100–6,000) were observed upon hospital admission.

The serologic test for measuring antibody to CMV was positive for immunoglobulin G (IgG) and negative for immunoglobulin M (IgM) and double negative for toxoplasmosis-specific antibodies (both IgG and IgM), treponema, hepatitis B surface antigen (HBsAg), and anti-HCV.

A male neonate was delivered by cesarean section due to persistent fetal tachycardia (gestational age 41 weeks; birth weight 3,595 g); cardiorespiratory adaptation at birth was good, the Apgar scores being 8 and 9 at 1 and 5 min, respectively.

Cord blood gas analysis, performed within 10 min after birth, showed normal pH, base excess (BE), and lactate. The neonate was eventually transferred in full isolation to the Neonatal Pathology Unit, separated from the mother.

Nasopharyngeal swab was first collected after having cleaned the baby, immediately after birth; it was tested with RT-PCR and was positive for the SARS-CoV-2 RdRp/N gene. It was then repeated 12 h after birth tested with RT-PCR and was positive for SARS-CoV-2 E and N genes.

Routine blood tests showed a normal number of white blood cells (16.55 × 10^3^/μl), PLT 357 × 10^3^/μl, normal PCR, elevated AST 143 UI/l (normal range 8–38), ALT 155 UI/l (12–41), gamma-glutamyltransferase (γGT) 126 UI/l (8–61), creatine phosphokinase (CPK) 917 UI/l (39–308), and lactate dehydrogenase (LDH) 1,216 UI/l (135–225). Blood culture was negative for bacteria or fungi.

During hospital stay, serial nasopharyngeal swabs were collected at 3, 7, and 10 days of life (DOL) and tested with RT-PCR and all were positive for the SARS-CoV-2 RdRp/N gene. At days 3 and 7 of life, SARS-CoV-2 was also searched and detected in the stools by RT-PCR for RdRp/N genes.

Routine blood tests, repeated at 3 DOL, confirmed the persistence of hypertransaminasemia [AST 96 U/l (24–90), ALT U/l 157 (7–33), elevated LDH 1072 U/l (225–600)] and γGT, CPK, and CRP within the normal range. Leucopenia (6,470 × 10^3^/μl) was also reported.

**Figure 1:**
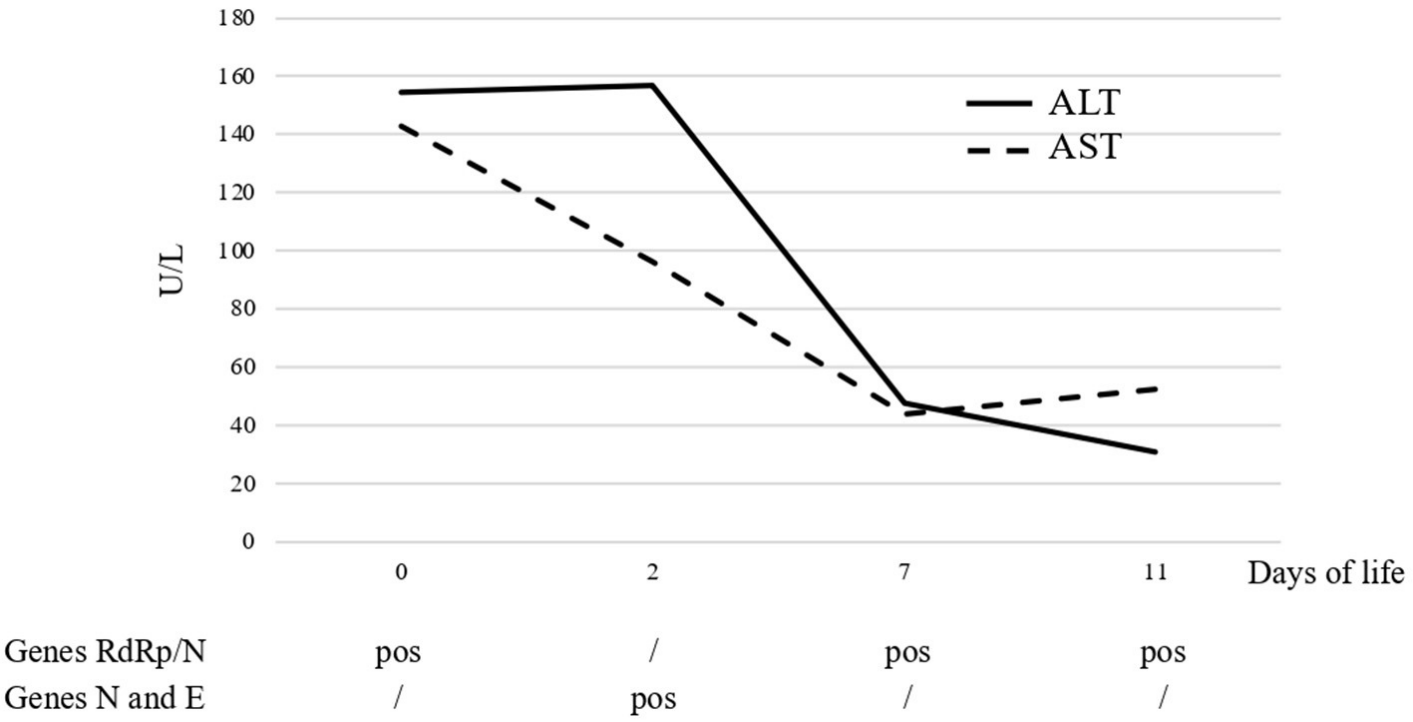
Trend course of serum liver enzymes and SARS-CoV-2-related genes detected by PCR on nasal swab.

The level of transaminases showed a progressive decrease starting from 7 DOL and was within the normal range by 10 DOL (Figure 1). White blood count cells were also normalized by 7 DOL. Abdomen ultrasound, performed at 4 DOL, reported a generalized increase in hepatic echogenicity.

Starting from 4 DOL, the neonate had mild diarrhea; therefore, probiotic was administered per os (Lactobacillus reuteri DSM 17938) and continued till discharge, with resolution of symptoms in 5 days.

The neonate was in good general clinical conditions during the entire hospital stay and was discharged from hospital with his mother at 12 DOL. The mother had a negative nasopharyngeal swab for SARS-CoV-2 10 days after parturition.

The first negative neonatal nasopharyngeal swab for SARS-COV-2 was performed at 18 DOL, at our pediatric referral center. Placental pathology report did not score the reported features of SARS-CoV-2 placentitis; RT-PCR results on placenta samples and amniotic fluid were not available.

## Discussion

We report liver involvement in a case of neonatal SARS-CoV-2 infection vertically acquired.

The detection of the virus by RT-PCR in nasopharyngeal swab at age <24 h defines the possibility of vertical transmission of SARS-CoV-2 (10). In addition, the neonate was born by C-section with intact amniotic membranes, thus suggesting a transplacental transfer of the virus (10). Compared to other viruses, SARS-CoV-2 is less placentotropic but can infect and cross the placenta (14) due to the binding to angiotensin-converting enzyme-2 (ACE2) receptors expressed in different feto-placental tissues (15).

Neonates infected by SARS-CoV-2 can alternatively be asymptomatic (45%) or develop symptomatic COVID-19 infection (55%); in this latter case, the most common symptoms include fever, GI, respiratory and neurological manifestations (16).

Liver injury was reported in COVID-19 adult and pediatric patients and can be attributed to different factors, including hypoxic–ischemic damage viral or drug-induced hepatocyte injury (17). In this case, liver injury was probably caused by a direct coronavirus-mediated mechanism, whose mechanistic details, albeit linked to ACE2 receptor expression in cholangiocytes and hepatocytes (59.7 and 2.6%, respectively), remain presently unknown. Alternative explanations for neonatal liver involvement (18) were likely excluded by the evidences that the neonate had a normal acid–base status and did not receive any medication before the first blood test examination. Maternal tests were negative for major congenital infections and neonatal blood cultures were negative; also, the Expanded Newborn Screening performed according to the Italian National Institute of Health neonatal screening program (19) excluded inherited metabolic disorders.

Unsurprisingly, we found the persistence of the virus in the feces until the last sample analyzed at 7 DOL. Emerging data suggest the prolonged presence of SARS-CoV-2 RNA in stool samples or rectal swabs even after the patients' respiratory specimens become negative (20), and much attention has been paid to the possibility of viral shedding from the GI tract and fecal–oral transmission.

Recent literature suggested that liver involvement, in case of SARS-CoV-2 infection, is possible, but to our knowledge, there are no reports that clearly described this association. A report by Kalamdani et al. (21) described the case of 12 newborns positive for SARS-CoV-2. Nine out of 12 newborns were tested for liver enzymes (AST and ALT) and reported a slight increase of median values of AST and ALT, lower compared to our case report, and in the range of normality considering the vast majority of cases. Moreover, authors reported just one value, and it is not specified in which day of life the blood drown was performed. Sisman et al. (22) described a case report of a preterm infant with intrauterine transmission of SARS-CoV-2 infection with slightly elevated AST (64 U/l range 10–35) and normal ALT; however, the trend of liver enzyme elevation was not described.

Zeng et al. (23) described a case series of 33 neonates born to mothers with COVID-19. Among them, three newborns tested positive for SARS-CoV-2. One had elevated AST (63 U/L) and ALT (88 U/L) and was born preterm (31 weeks GA). Moreover, his clinical course was complicated by respiratory distress syndrome, pneumonia, and suspected sepsis; thus, other causes of elevated transaminases were plausible.

We presented a case of a well-documented neonatal infection, describing AST and ALT trend over time and exclusion of other causes of hepatic involvement. This clinical case suggests that possible liver damage should be sought in all newborns born to COVID-19-positive mothers, regardless of the clinical condition. However, further studies are needed to confirm our observations. Longer follow-up and prospective studies are needed to determine the real impact of SARS-CoV-2 virus in the liver.

## Data Availability Statement

The data analyzed in this study is subject to the following licenses/restrictions: Not applicable. Requests to access these datasets should be directed to gianluca.terrin@uniroma1.

## Ethics Statement

Ethical review and approval was not required for the study on human participants in accordance with the local legislation and institutional requirements. Written informed consent to participate in this study was provided by the participants' legal guardian/next of kin. Written informed consent was obtained from the individual(s), and minor(s)' legal guardian/next of kin, for the publication of any potentially identifiable images or data included in this article.

## Author Contributions

IS and MGC designed the study and interpreted the data. IS, MGC, AM, LD, FN, MB, RC, DR, AT, IP, FP, and CA provided the patients' and relatives' data and contributed to the first draft. MGC, RB, and GT interpreted the data and critically revised the last version of the manuscript. All authors contributed to the article and approved the submitted version.

## Conflict of Interest

The authors declare that the research was conducted in the absence of any commercial or financial relationships that could be construed as a potential conflict of interest.

## Publisher's Note

All claims expressed in this article are solely those of the authors and do not necessarily represent those of their affiliated organizations, or those of the publisher, the editors and the reviewers. Any product that may be evaluated in this article, or claim that may be made by its manufacturer, is not guaranteed or endorsed by the publisher.
